# The Frog Sign Revisited

**DOI:** 10.19102/icrm.2022.13101

**Published:** 2022-10-15

**Authors:** Christopher J. Ho, Reginald T. Ho

**Affiliations:** ^1^Department of Biology, James Madison University, Harrisonburg, VA, USA; ^2^Department of Medicine, Division of Cardiology, Thomas Jefferson University Hospital, Philadelphia, PA, USA

**Keywords:** Frog sign, right atrial pressure, typical atrioventricular nodal re-entrant tachycardia

## Abstract

The frog sign is a classic physical examination finding of typical atrioventricular nodal re-entrant tachycardia. We present the case of a 78-year-old man with recurrent, symptomatic supraventricular tachycardia referred for ablation in whom the frog sign was observed during physical examination.

The frog sign is a classic physical examination finding of typical atrioventricular (AV) nodal re-entrant tachycardia (AVNRT) due to right atrial contraction against a closed tricuspid valve resulting in a reflux of blood into the superior vena cava and jugular veins.^1^ Because of transmitted ventricular waveforms during right atrial pressure (RAP) recordings, RAP measurements during ventricular pacing maneuvers might further facilitate the diagnosis of AVNRT.

A 78-year-old man with recurrent, symptomatic supraventricular tachycardia (SVT) was referred for SVT ablation **(Figure 1)**. Upon presentation, he was in tachycardia, and the frog sign was observed on physical examination. An electrophysiologic study with RAP recordings was performed. Frequent non-sustained atrial tachycardia repeatedly induced an “A-on-V” tachycardia that was terminated by late-coupled His-refractory atrial premature depolarizations (*), excluding junctional tachycardia **(Figure 2)**.^2^ Characteristic low-pressure sinus waveforms transitioned to higher-pressure (peak, 25 mmHg) fused ventriculoatrial “cannon waves” (VA = 0 ms) during tachycardia. During onset of entrainment from the ventricle, atrial waveforms (↓) were dissociated from the ventricle within the transition zone, causing a drop in RAP, but then became fixed with the ventricle (VA = 112 ms) following acceleration of the atrial rate to the pacing cycle length (410 ms) after the third fully paced ventricular complex (hemodynamic ∆VA [VA_(V entrainment)_ – VA_(SVT)_] = 112 ms [>85 ms]) **(Figure 3)**.^3,4^ Termination of entrainment revealed an “AV” response with a long hemodynamic post-pacing interval (549 ms) that exceeded the tachycardia cycle length by 131 ms (>115 ms), followed by resumption of cannon waves.^5,6^ Slow pathway ablation rendered the AVNRT non-inducible.

Besides cannon waves, hemodynamic post-pacing interval and ∆VA intervals derived from RAP waveforms can be useful for the diagnosis of typical AVNRT.

## Figures and Tables

**Figure 1:**
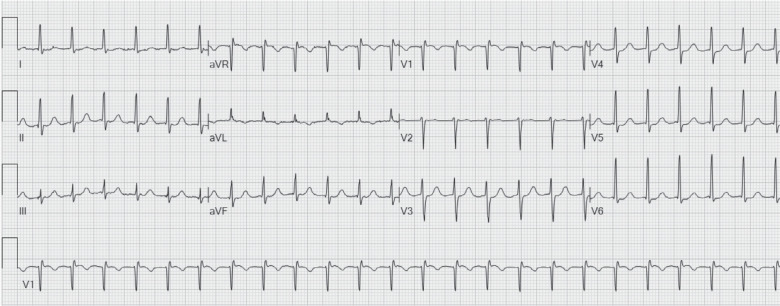
Twelve-lead electrocardiogram of supraventricular tachycardia.

**Figure 2:**
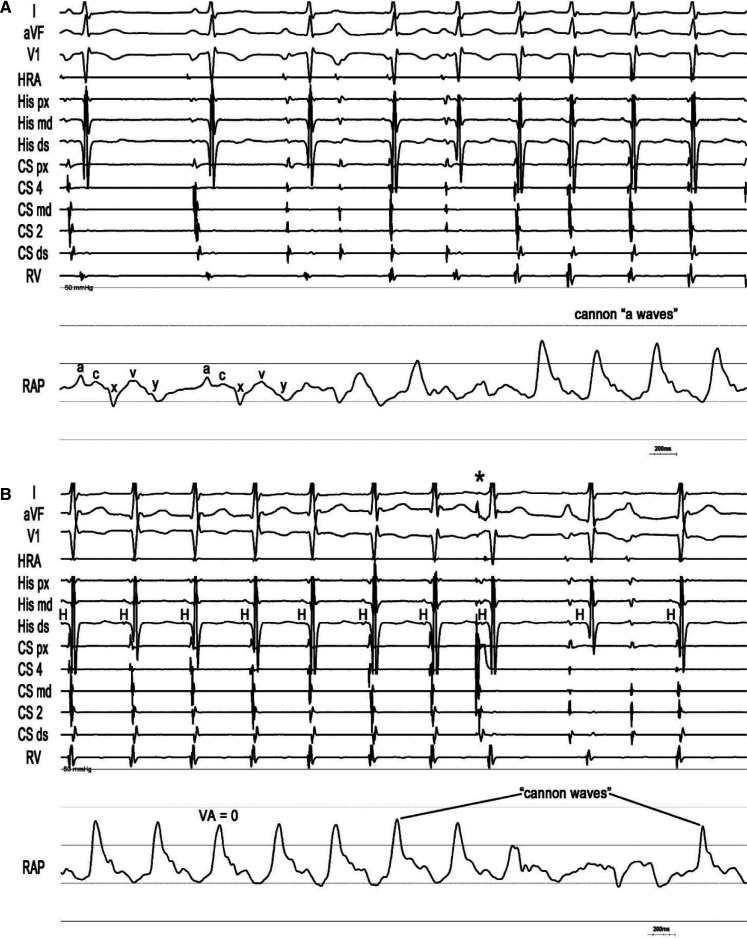
Onset **(A)** and termination **(B)** of typical atrioventricular nodal re-entrant tachycardia with right atrial pressure (RAP) recordings. The positive a-wave represents atrial contraction that actively fills the right ventricle in end-diastole.^7^ The positive c-wave results from the closure and bulging of the tricuspid valve into the right atrium during early systole. The positive v-wave reflects a passive increase in RAP as the right atrium fills in late systole and early diastole. The x descent reflects atrial relaxation in the final phase of ventricular systole. The y descent results from the opening of the tricuspid valve and passive ventricular filling in early diastole. *Abbreviations:* CS, coronary sinus; HRA, high right atrium; RAP, right atrial pressure; RV, right ventricle; VA, ventriculoatrial. *His-refractory atrial premature depolarization.

**Figure 3:**
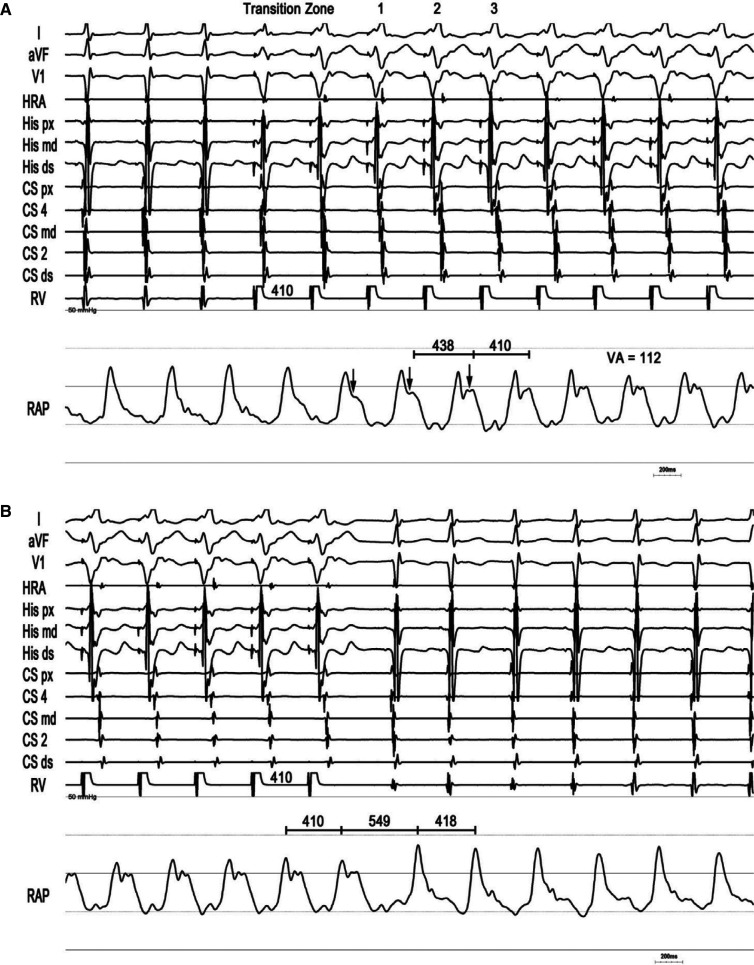
Onset **(A)** and termination **(B)** of entrainment of typical atrioventricular nodal re-entrant tachycardia from the ventricle with right atrial pressure recordings. *Abbreviations:* ↓, atrial pressure waveforms dissociated from ventricular waveforms; CS, coronary sinus; HRA, high right atrium; RAP, right atrial pressure; RV, right ventricle; VA, ventriculoatrial.

## References

[1] Gursoy S, Steurer G, Brugada J, Andries E, Brugada P (1992). Brief report: The hemodynamic mechanism of pounding in the neck in atrioventricular nodal reentrant tachycardia. N Engl J Med.

[2] Padanilam BJ, Manfredi JA, Steinberg LA, Olson JA, Fogel RI, Prystowsky EN (2008). Differentiating junctional tachycardia and atrioventricular node re-entry tachycardia based on response to atrial extrastimulus pacing. J Am Coll Cardiol.

[3] Al Mahammeed ST, Buxton AE, Michaud GF (2010). New criteria during right ventricular pacing to determine the mechanism of supraventricular tachycardia. Circ Arrhythm Electrophysiol.

[4] Dandamudi G, Mokabberi R, Assal C (2010). A novel approach to differentiating orthodromic reciprocating tachycardia from atrioventricular nodal reentrant tachycardia. Heart Rhythm.

[5] Knight B, Zivin A, Souza J (1999). A technique for the rapid diagnosis of atrial tachycardia in the electrophysiology laboratory. J Am Coll Cardiol.

[6] Michaud GF, Tada H, Chough S (2001). Differentiation of atypical atrioventricular node re-entrant tachycardia from orthodromic reciprocating tachycardia using a septal accessory pathway by the response to ventricular pacing. J Am Coll Cardiol.

[7] Applefeld MM,  Walker HK, Hall WD, Hurst JW (1990). The jugular venous pressure and pulse contour. Clinical Methods: The History, Physical, and Laboratory Examinations.

